# QTc prolongation prior to angiography predicts poor outcome and associates significantly with lower left ventricular ejection fractions and higher left ventricular end-diastolic pressures

**DOI:** 10.5830/CVJA-2012-060

**Published:** 2012-11

**Authors:** Pieter Van Der Bijl, Marshall Heradien, Anton Doubell, Paul Brink

**Affiliations:** Division of Cardiology, Department of Medicine, Faculty of Health Sciences, Stellenbosch University and Tygerberg Academic Hospital, Western Cape, South Africa; Division of Cardiology, Department of Medicine, Faculty of Health Sciences, Stellenbosch University and Tygerberg Academic Hospital, Western Cape, South Africa; Division of Cardiology, Department of Medicine, Faculty of Health Sciences, Stellenbosch University and Tygerberg Academic Hospital, Western Cape, South Africa; Division of Cardiology, Department of Medicine, Faculty of Health Sciences, Stellenbosch University and Tygerberg Academic Hospital, Western Cape, South Africa

**Keywords:** QT prolongation, sudden death, coronary artery disease

## Abstract

**Background:**

QT prolongation on the surface ECG is associated with sudden cardiac death. The cause of QT prolongation in ischaemic heart disease (IHD) patients remains unknown, but may be due to a complex interplay between genetic factors and impaired systolic and/or diastolic function through as yet unexplained mechanisms. It was hypothesised that QT prolongation before elective coronary angiography is associated with an increased mortality at six months.

**Methods:**

Complete records of 321 patients who underwent coronary angiography were examined for QT interval corrected for heart rate (QTc), left ventricular ejection fraction (LVEF), left ventricular end-diastolic pressure (LVEDP) and known ischaemic heart disease risk factors. Patients were designated long QTc (LQTc) when they had prolonged QTc intervals or normal QTc (NQTc) when the QTc interval was normal. Patients with atrial fibrillation, bundle branch blocks, no ECG in the 24 hours before angiography, or a creatinine level > 200 μmol/l were excluded. Survival was determined telephonically at six months.

**Results:**

Twenty-eight per cent of the total population had LQTc. During follow up, 15 patients (4.7%) died suddenly, 73% of whom had a LQTc. LQTc was significantly associated with mortality (LQTc 12% vs NQTc 1.7%; *p* < 0.01), and with lower but normal LVEF (LQTc 52.9 ± 15.4% vs NQTc 61.6 ± 13.6%; *p* < 0.01), higher LVEDP at LVEF > 45% (LQTc 19.2 ± 9.0 mmHg vs NQTc 15.95 ± 7.5 mmHg; *p* < 0.05), hypercholesterolaemia and a negative family history of IHD.

**Conclusion:**

In patients with sinus rhythm and normal QRS width, QTc prolongation before coronary angiography predicted increased mortality at six months. QTc also associated strongly with left ventricular systolic and diastolic dysfunction, hypercholesterolaemia and a negative family history of IHD.

## Abstract

The QT interval represents ventricular electrical depolarisation and repolarisation on the surface ECG. It increases with age and varies with gender, time of day, season of the year and heart rate.^1^-^3^ Bazett’s formula is frequently used to correct the QT interval for heart rate, yielding the QTc interval.^4^,^5^

A prolonged QTc interval (LQTc) is a manifestation of a complex interplay between genetic and environmental factors, and is a risk factor for life-threatening dysrhythmias and sudden death. The *forme fruste* of QT prolongation is congenital long QT syndrome (LQTS), an inherited cardiac ion-channel disease associated with syncope, malignant ventricular tachydysrhythmias and sudden cardiac death. LQTS, by exclusion, occurs in structurally normal hearts.^6^

Recently, polymorphisms in the gene for NOS1AP, which regulates nitric oxide production and thus coronary perfusion, have been shown to prolong cardiac repolarisation.^7^-^9^ Ethnic differences in QT interval have also been reported, LQTc comprising a higher risk among black than white subjects.^10^

QT prolongation is an independent prognosticator of cardiac and all-cause mortality, especially in the context of cardiovascular disease.^11^-^13^ In addition, it is associated with an increased 10-year risk of ischaemic heart disease (IHD) and sudden death in the general population.^14^

The fact that interventional cardiologists are more interested in the ‘ST’ segment than the ‘QTc’ is underscored by the paucity of QTc studies peri-angiography. In only one study has an LQTc in the period immediately before coronary angiography been directly correlated with outcome, but the authors described QT peak interval instead of QTc.^15^ LQTc in the presence of coronary artery disease increases the risk of sudden cardiac death by a factor of five.^16^

IHD is a cause of systolic and diastolic dysfunction of the left ventricle, and both of these are independent predictors of mortality.^17^,^18^ QT prolongation is not only inherited, but also linked to cardiac hypertrophy, as frequently observed in hypertensive heart disease.^19^ However, no reference to an association between left ventricular systolic and diastolic dysfunction and QTc could be identified in the literature. Additionally, the length of the QTc interval appears to be directly related to the number of large coronary arteries that are diseased.^20^

QT prolongation may also reflect autonomic neuropathy in patients with diabetes mellitus.^21^,^22^ It does not, however, provide a reliable measure of the degree of autonomic neuropathy.^23^ Although smoking has been associated with a LQTc, it has not been shown to be an independent cause of a LQTc.^24^ No clear link is evident in the literature between hypercholesterolaemia and QTc interval.

The purpose of our study was to evaluate LQTc as an independent prognostic indicator with regard to mortality and systolic and diastolic dysfunction in the context of IHD. Furthermore, we endeavoured to assess, in a state hospital setting in the Western Cape, whether LQTc correlated with triple-vessel coronary artery disease (TVCAD), or was significantly associated with hypercholesterolaemia, diabetes mellitus, smoking, hypertension or a family history of IHD.

## Methods

The study was approved by the Committee for Human Research, Faculty of Health Sciences, Stellenbosch University. All patients signed informed consent before coronary angiography. All data were collected and recorded as part of routine clinical care.

This was a single-centre, prospective study, enrolling a cohort of patients who were eligible for coronary angiography from 2006 to 2009 at Tygerberg Academic Hospital. Due to the time limit imposed by the authors collecting data, rotating through the coronary care unit, not all eligible patients could be enrolled. Patients with atrial fibrillation, bundle branch blocks, no ECG in the 24 hours before angiography, or renal failure (creatinine ≥ 200 μmol/l) were excluded from the study.^25^,^26^

QTc intervals were recorded on the last ECG taken during the 24 hours before coronary angiography, and designated long QTc (LQTc) or normal QTc (NQTc). The primary outcome was six-month survival. Secondary outcomes included correlation of QTc intervals with left ventricular ejection fraction (LVEF), left ventricular end-diastolic pressure (LVEDP), TVCAD, diabetes mellitus, smoking, systemic hypertension, a family history of IHD and dyslipidaemia.

## Measurement of QT interval

As the accuracy of automated measurements of the QT interval is questionable, partly because of inconsistency between manufacturers in terms of the digital algorithms employed in various instruments, the QT intervals were measured manually.^5^ These values were then compared with the algorithm-based ones.

The QT interval was measured from the start of the QRS complex to the end of the T wave, using a Digimatic® digital caliper (0.01-mm scale) with an autostopper (Mitutoyo Corporation, Mita, Japan). When a T wave was biphasic, or nearly so, the QT interval was measured to include the final return to baseline. No U waves were encountered, and therefore no decision as to the endpoint of measurement in such instances had to be taken. The digitally calculated heart rate was obtained from the ECG, and the QTc was computed by means of Bazett’s formula.^4^,^5^

## Definition of parameters and endpoints

The QTc was considered prolonged if > 440 ms in males and > 460 ms in females. The follow-up period started on the day of coronary angiography (day 0), and ended after six months (day 180) had elapsed. Survival was determined telephonically at the end of six months. The LVEF (as an index of systolic function) was determined either by ventriculography at the time of coronary angiography or, in the case of ventriculography not having been performed, by transthoracic or trans-oesophageal echocardiography. The LVEDP (as an index of diastolic function) was measured at the time of coronary angiography via a fluid-filled endocardiac catheter.

TVCAD was defined as a > 70% stenosis of all three of the following vessels: the right main coronary artery, the left circumflex artery and the left anterior descending coronary artery or the right main coronary artery, in conjunction with the left main stem, as assessed at the time of coronary angiography. The interventional cardiologist decided how to treat stenosed coronaries, i.e. medically, percutaneously or surgically, and the investigators were blinded to subsequent treatment.

Diabetes mellitus was defined as symptoms of diabetes plus a random blood glucose concentration of ≥ 11.1 mmol/l, or fasting plasma glucose of ≥ 7.0 mmol/l. This accords with the American Diabetes Association clinical practice recommendations of 2006.^27^ In addition, patients were considered diabetic if they were taking anti-diabetic medication.

Smoking was defined as the use of any tobacco on a daily basis at any stage of the patient’s life. Systemic hypertension was defined as a blood pressure recording of ≥ 140/90 mmHg as per National Cholesterol Education Program Adult Treatment Panel III Guidelines of 2004.^28^ A family history of IHD was considered positive if it was present in a male first-degree relative of ≤ 55 years, or in a female first-degree relative of ≤ 65 years.^28^

Hypercholesterolaemia was defined as a total, non-fasting serum cholesterol level > 5 mmol/l. The non-fasting nature was accepted as a result of the short period of hospitalisation of most patients undergoing angiography.

## Statistical analysis

Data were analysed by the Stellenbosch University Centre for Statistical Consultation (CSC) using STATISTICA version 9 (StatSoft® Inc, Tulsa, OK, USA, 2009). The Kaplan–Meier method was used to create survival curves for NQTc and LQTc groups and a log-rank test was used to compare the two curves and generate a *p*-value. NQTc and LQTc groups were compared with regard to LVEF, LVEDP and serum cholesterol with a Mann–Whitney *U* test. Categorical data (diabetes mellitus, smoking, hypertension and family history of IHD) were tested for an association with LQTc using a chi-square test. Statistical significance was defined as *p* < 0.05.

## Results

**Fig. 1:**
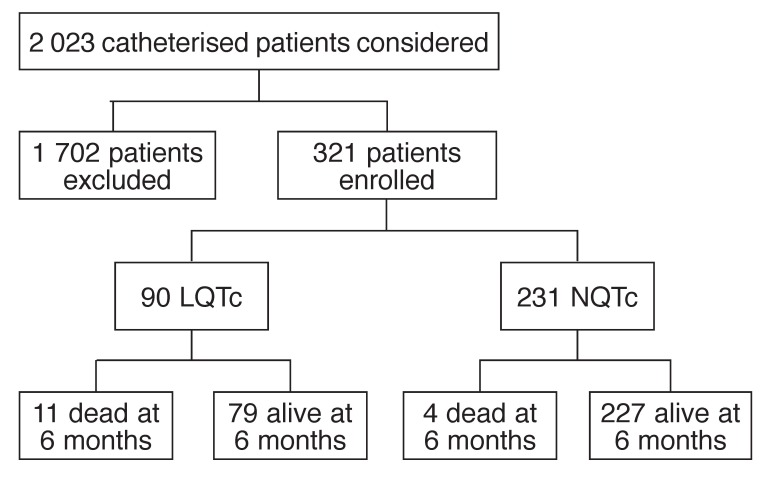
Allocation of study subjects.

Of the 2 023 patients who were catheterised, 321 were enrolled (80.2% Coloured, 18.8% white, 1.0% black) (mean age 56 ± 12 years; range 24–84) (Fig. 1). One hundred and sixteen patients (36%) were female (mean age 57 ± 10 years; range 29–78) and 205 (64%) were male (mean age 55 ± 12 years; range 24–85) (*p* = 0.168). Ninety patients (28%) had LQTc.

**Fig. 2:**
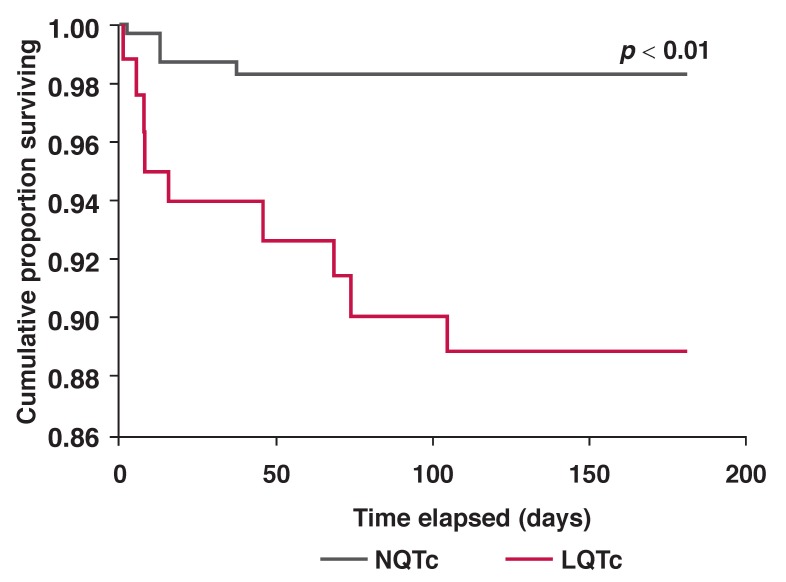
Cumulative proportion of patients surviving versus time elapsed (Kaplan–Meier).

At six months, 15 (4.7%) of the total population had died, 73% of whom had a LQTc. Eleven patients (12%) died in the LQTc group and four (1.7%) in the NQTc group (*p* < 0.01) (Fig. 2). The hazard ratio was 10.16 (95% CI: 2.91–35.44).

**Fig. 3:**
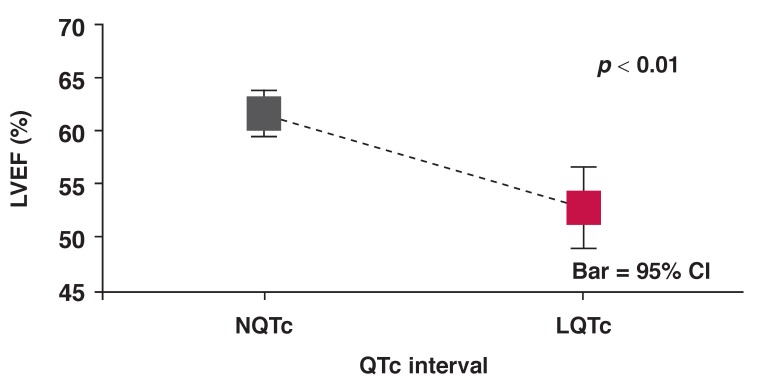
Mean LV EF versus QTc interval for NQTc and LQTc groups of patients.

**Fig. 4:**
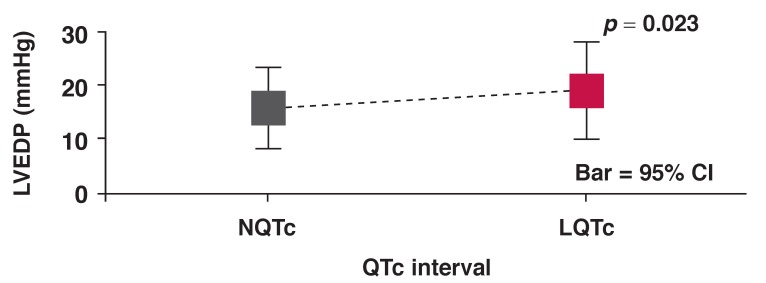
Mean LV EDP (LV EF > 45%) versus QTc interval for NQTc and LQTc groups of patients.

**Fig. 5:**
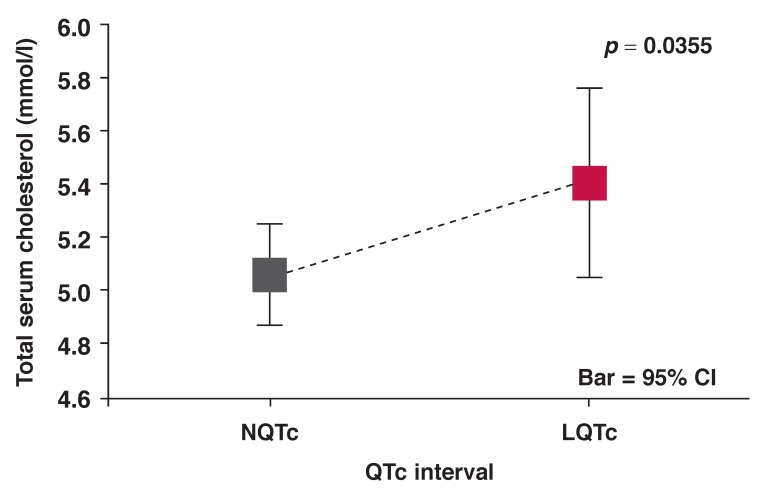
Mean total serum cholesterol versus QTc interval for NQTc and LQTc groups of patients.

LQTc patients had normal LVEF but lower than that of the NQTc cohort (LQTc: 52.9 ± 15.4% vs NQTc: 61.6 ± 13.6%; *p* < 0.01) (Fig. 3) and higher LVEDP at LVEF > 45% (LQTc: 19.2 ± 9.0 mmHg vs NQTc: 15.9 ± 7.5 mmHg; *p* < 0.05) (Fig. 4). The LQTc cohort also had significantly higher serum cholesterol levels than the NQTc cohort (Fig. 5).

A family history of IHD was significantly less common among those with LQTc (*p* = 0.045) (Table 1). No association between LQTc and diabetes mellitus, smoking or hypertension could be detected. No association could be demonstrated between TVCAD and NQTc or LQTc (*p* = 0.96).

**Table 1.** Association Between LQTc And NQTc Groups Of Patients With Regard To Major Risk Factors For IHD

*Risk factor present*	*Yes, n (%)*	*No, n (%)*	*Total,* n	p*-value*
Diabetes mellitus				
NQTc	62 (29)	149 (71)	211	
LQTc	29 (33)	59 (67)	88	0.54
Total *n*	91	208	299	
Smoking				
NQTc	116 (56)	92 (44)	208	
LQTc	55 (64)	31 (36)	86	0.19
Total *n*	171	123	294	
Hypertension				
NQTc	157 (74)	55 (26)	212	
LQTc	70 (80)	17 (20)	87	0.23
Total *n*	227	72	299	
Family history				
NQTc	74 (36)	134 (64)	208	
LQTc	21 (24)	67 (76)	88	0.045*
Total *n*	95	201	296	

*Statistically significant association.

## Discussion

This study provides further evidence that, regardless of coronary revascularisation, QT prolongation before coronary angiography associated significantly with increased mortality at six months, lower LVEF and higher LVEDP. With regard to IHD risk factors, LQTc patients had higher serum cholesterol values and rarely a family history of IHD.

Traditionally, QT prolongation was seldom regarded by interventional cardiologists to be clinically useful, but it has recently been shown that the risk for coronary stenosis increases by 33 to 41% for every 20-ms QTc interval prolongation.^29^ The ST segment, on the other hand, which forms an integral part of the QT interval, is considered a better marker of underlying coronary artery disease. Interventional cardiologists rely heavily on ST segment shifts during exercise and recovery to identify those patients who need diagnostic coronary angiography. Little attention is given to the QTc interval, especially if it has to be measured or calculated manually.

Once coronary angiography has been performed, stents have been inserted and dual anti-platelet therapy (DAT) and beta-blockers have been prescribed, patients are often seen only weeks later for follow up at the outpatient clinic, where they are assessed by a junior colleague. The routine ECG is studied for new Q waves and cardiac arrhythmias. The blood pressure is checked, drug compliance with DAT is reiterated, and the patient is given a six-month follow-up appointment. Our data suggest that more than 10% of patients with QTc prolongation prior to coronary angiography will not return for their six-month appointment because they have died suddenly.

The association of LQT with a lower but normal LVEF is interesting. While there is no internationally agreed threshold of a ‘low’ LVEF, it has been characterised in a report of the American Society of Echocardiography and the European Association of Echocardiography by gradation: mild (45–54%), moderate (30–44%) and severe (< 30%) left ventricular dysfunction.^30^ Only one study reported an association between LQTc and impaired LV function.^31^ The authors also reported that the QTc interval increased significantly from one- to three-vessel disease. We did not find this correlation between TVCAD and LQTc in our study.

The mechanism by which a decreased LVEF results in QT prolongation is unknown, but may involve ion-channel remodelling and/or intracellular calcium transport.^32^ LVEF is frequently used to prognosticate patients who have suffered a myocardial infarction or who have dilated cardiomyopathy. It is generally accepted that patients with an impaired systolic function have a higher risk for sudden cardiac death, presumably due to malignant ventricular tachydysrhythmias. In the Sudden Cardiac Death in Heart Failure Trial (SCD-HeFT), implantable cardioverter-defibrillator (ICD) therapy reduced all-cause mortality by 23% compared with placebo.^33^ Interestingly, amiodarone, a commonly used anti-arrhythmic agent that also prolongs the QT interval, was ineffective to prevent sudden death in these patients.

The association of LQT with an elevated LVEDP (at a normal LVEF – frequently defined as > 45% , and used for the purpose of this study) is striking. Diastolic dysfunction is a relatively new concept when compared with LVEF. The commonest cause of diastolic dysfunction is hypertensive heart disease. Often these patients have thicker, stiffened left ventricles with good systolic function, but impaired relaxation and compliance. Again, exactly how diastolic dysfunction in IHD patients is associated with QT prolongation remains unknown, but recently an association was found between down-regulation of the hERG gene and QT prolongation in rats with cardiac hypertrophy.^34^

Ion-channels are embedded in a phospholipid bi-layer primarily composed of cholesterol esters. Both congenital LQTS and familial hypercholesterolaemia are more common in South Africans of European descent. Co-segregation of ion-channel disease and hypercholesterolaemia has not yet been described in humans, but in Langendorff-perfused rabbit hearts, hyperlipidaemia led to significant QT prolongation compared with normocholesterolaemia, which can be reversed by administering simvastatin.^35^

## Study limitations

This single-centre study in a state hospital setting may be prone to selection bias due to the fact that patients were enrolled only during rotations of the authors collecting the data through the coronary care unit. However, all eligible patients were enrolled during these intervals, leading us to believe that the cohort was truly representative.

More than 80% of the studied population were of mixed racial ancestry. One should therefore be careful to draw conclusions about race and QT prolongation.

Prescribed medications were not checked and these may well have prolonged the QT interval after discharge. However, this study addressed the relationship between QTc prior to coronary angiography and mortality at six months.

The effects of coronary revascularisation on QTc were also not investigated but it was assumed that significant coronary stenosis would have been treated appropriately by the interventional cardiologist. Mortality in the LQT cohort remained high regardless of coronary revascularisation. Follow up was relatively short owing to the vast extent of the geographical catchment area of the hospital.

Genetic screening was also not performed on the study patients. Diastolic pressure was used as an indicator of diastolic function; however, echocardiographic parameters of diastolic function were not assessed.

## Conclusion

This is the first description of LQTc in a cohort of IHD patients in a South African setting. The study confirms that QTc, which can be determined by a simple, non-invasive, inexpensive method, is an index of subsequent sudden death in patients who undergo coronary angiography for suspected IHD.

QTc prolongation before coronary angiography is also a reflection of systolic and diastolic dysfunction (in the context of normal systolic function) of the left ventricle, both of which are independent predictors of mortality rate. Furthermore, LQTc correlates with hypercholesterolaemia and a negative family history of IHD.
